# Association of logarithmic lymphocyte-albumin product with active tuberculosis in children and adolescents

**DOI:** 10.3389/fnut.2026.1781667

**Published:** 2026-04-14

**Authors:** Jinyu Chen, Bin Deng, Yan Wang, Shenjie Tang, Dongmei Wang, Lei Chen, Qi An

**Affiliations:** 1 Department of Tuberculosis, Public Health Clinical Center of Chengdu, Chengdu, Sichuan, China; 2 Beijing Chest Hospital, Capital Medical University, Beijing, China

**Keywords:** active tuberculosis, albumin, biomarker, children and adolescents, immune-nutritional biomarker, lymphocyte

## Abstract

**Background:**

Active tuberculosis (ATB) in children and adolescents remains a major diagnostic challenge. Inflammation, nutrition, and immune status are closely linked to tuberculosis (TB) progression. We therefore proposed a novel biomarker, the logarithmic lymphocyte-albumin product (logLAP), integrating immune and nutritional indicators, and investigated its association with ATB in children and adolescents.

**Methods:**

This retrospective study included 1,080 participants aged <18 years. Participants were classified into ATB (*n* = 904) and non-TB (*n* = 176) groups. LAP was calculated as lymphocyte × albumin, and logLAP was derived as log(LAP). Discriminatory ability was evaluated using receiver operating characteristic (ROC) curves. Logistic regression models were applied to assess the association between logLAP and ATB. Restricted cubic spline (RCS) analyses and stratified analyses were conducted to explore nonlinear relationships and subgroup differences.

**Results:**

Compared with non-TB controls, ATB patients exhibited significantly lower logLAP levels (4.07 ± 0.55 vs. 4.39 ± 0.47, *p* < 0.001). In ROC analysis, LAP achieved the higher area under the curve (AUC = 0.6955) than neutrophil-lymphocyte ratio (NLR), neutrophil-albumin ratio (NAR), platelet-lymphocyte ratio (PLR), systemic immune-inflammation index (SII) and prognostic-nutritional index (PNI). The clinical diagnostic cut-off value for logLAP was 4.23, with a sensitivity of 60% and a specificity of 74%. Logistic regression models revealed the third tertile (Q3) of logLAP showed a strong inverse association with ATB (OR = 0.33, 95% CI: 0.18–0.59, *p* < 0.001) after full adjustment. RCS analyses with four knots revealed a significant non-linear relationship between logLAP and ATB (*p* for non-linearity = 0.021), confirming a threshold effect at logLAP = 3.48. Stratified analyses indicated consistent associations across most subgroups, while logLAP showed limited associations in children aged 0–7 years and those with severe immune dysfunction (CD4+/CD8+ T cell counts below the reference range). The AUC of logLAP demonstrated a clear advantage over traditional biomarkers; however, its diagnostic performance remained relatively limited. This suggests that logLAP is more suitable as a supplementary tool and should be used in conjunction with other clinical or laboratory indicators.

**Conclusion:**

As a biomarker integrating immune-nutritional status, logLAP exhibits a robust inverse association with ATB infection in children and adolescents aged ≥8 years with normal or moderately impaired immune function, supported by comprehensive analyses.

## Graphical abstract

**Figure:**
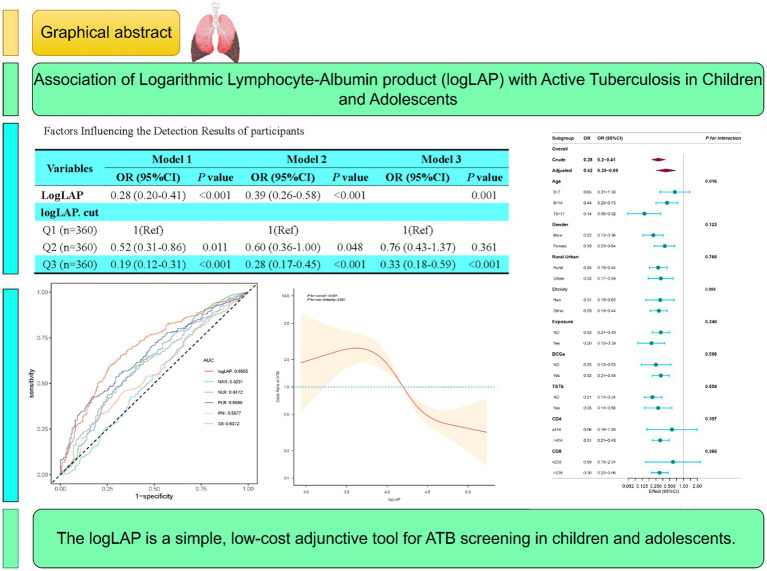

## 1 Introduction

Tuberculosis (TB) remains the leading cause of death worldwide attributed to a single pathogen and ranks among the top 10 global causes of death. According to the *Global Tuberculosis Report 2025*, there were 10.7 million TB infections globally in 2024, with a case fatality rate of 11.5% (1). Children and adolescents account for 11% of all TB cases worldwide, with a fatality rate of approximately 15%, which is higher than the overall average (1). Active tuberculosis (ATB) in children and adolescents is often characterized by atypical clinical presentations and paucibacillary disease, coupled with the difficulty in obtaining sputum samples. These factors contribute to delayed diagnosis, and increased risk of disease progression, making early detection, diagnosis, and treatment crucial for ending TB (2, 3). Among children suspected of having TB, only about 6.8% test positive on acid-fast bacillus (AFB) smear microscopy (4). The culture of *Mycobacterium tuberculosis* (MTB), requiring several weeks, yields a positivity rate of only 30–40% (4). Immunodiagnostic tools such as the tuberculin skin test (TST) and interferon-gamma release assays (IGRAs) may cross-react with Bacillus Calmette–Guérin (BCG) vaccination, affecting their accuracy (2). In 2022, the WHO’s consolidated guidelines on TB in children and adolescents recommended the Xpert MTB/RIF ultra-rapid molecular assay as an initial diagnostic test (2). However, Xpert MTB/RIF shows suboptimal sensitivity (22–53%) when applied to non-respiratory samples, such as oral swabs (2, 5). Moreover, its high cost and dependence on specialized laboratory infrastructure present challenges for implementation in resource-limited regions. Therefore, there is an urgent need for novel, simple, and rapid indicators with good discriminatory ability to support clinical assessment and reduce diagnostic delays in children and adolescents with ATB.

In recent years, complete blood count (CBC)-derived biomarkers have attracted growing interest in clinical diagnostics, as they are characterized by low cost and straightforward calculation procedures. The neutrophil-to-lymphocyte ratio (NLR), platelet-to-lymphocyte ratio (PLR), and systemic immune-inflammation index (SII) have been explored as biomarkers of systemic inflammation and immune dysregulation in individuals with ATB (6–8). For children and adolescents with ATB, studies on NLR in pediatric TB have yielded variable results, with AUCs ranging from 0.72 to 0.82 depending on control groups (9, 10), while PLR showed poor performance in predicting latent TB in adolescents (AUC = 0.56) (11). SII has not been validated in any pediatric ATB cohort to date (8). The prognostic nutritional index (PNI), which combines albumin levels and lymphocyte count, reflects the interplay between nutrition and immunity and has been associated with disease severity and prognosis in TB (12, 13). The neutrophil-to-albumin ratio (NAR), integrating both inflammatory and nutritional status, has been used to assess prognosis in conditions such as Myasthenia Gravis and depression (14, 15). While several studies have explored the association between these biomarkers and TB, their diagnostic performance in the children and adolescents population remains inconsistent or under-investigated. The distinct immunological and developmental characteristics of children and adolescents may influence the utilities of these biomarkers.

TB pathogenesis and clinical progression are tightly linked to the immune and nutritional status of the host. Lymphocytes are central to the adaptive immune response against MTB and are essential for acquired anti-TB immunity. During ATB infection, their numbers decrease, creating favorable conditions for MTB proliferation within the host (16). The WHO, in its “End TB Strategy,” recommends that all individuals with TB should have their nutritional status assessed (17). Nutrients are closely linked to enhancing the body’s defense against intracellular infections such as MTB, and malnutrition increases the likelihood of progression from latent to ATB (18). Nutrients also possess immunomodulatory properties, regulating inflammatory and infectious processes (19). Serum albumin serves as a robust marker of both nutritional status and systemic inflammation (20, 21). Children and adolescents are particularly vulnerable to malnutrition, which can impair cellular immunity and exacerbate TB severity (18, 22). Therefore, we hypothesize that composite markers integrating immune and nutritional parameters may offer better diagnostic value for ATB in children and adolescents.

Given the research gaps in pediatric ATB-specific immune-nutritional biomarkers, lymphocyte–albumin product (LAP) represents a combined biomarker of immune capacity and nutritional status. Calculated as the product of lymphocyte count and albumin level, LAP may better reflect the synergistic relationship between cellular immunity and nutrition. Logarithmic transformation of LAP (logLAP) can further normalize its distribution and improve its analytical performance. To date, LAP/logLAP as a new biomarker has not been explored in any disease. We hypothesize that logLAP may be associated with ATB status in children and adolescents and could provide better diagnostic performance compared to existing inflammatory ratios. In this study, we aimed to investigate the relationship between logLAP and the risk of ATB infection in children and adolescents. We assessed the discriminatory ability of LAP by comparing it with established biomarkers such as NLR, NAR, PLR, SII and PNI. Furthermore, we evaluated the independent association between logLAP and ATB infection using logistic regression, adjusting for key demographic, clinical, and immunological confounders. Finally, we utilized restricted cubic splines (RCS) and stratified analyses to explore potential nonlinear relationships between logLAP and ATB infection, as well as the stability of diagnostic performance across different population subgroups. Through these analyses, we aimed to establish logLAP as a simple biomarker associated with ATB infection in children and adolescents, potentially offering a new auxiliary indicator for clinical assessment of ATB in this population.

## 2 Methods

### 2.1 Study design and participants

This retrospective study enrolled children and adolescents aged <18 years who were hospitalized at the Public Health Clinical Medical Center of Chengdu between April 2022 and December 2024. All included patients had been evaluated for either ATB or non-TB diseases. Participants were divided into the ATB group and non-TB group. The ATB group was further classified into etiologically confirmed TB (E-TB) and clinically diagnosed TB (C-TB). E-TB was defined as positive molecular biology tests for MTB or positive MTB culture, combined with radiological manifestations consistent with TB. C-TB was defined as radiological findings consistent with TB (with or without clinical symptoms) and a positive response to anti-TB treatment. The non-TB group included patients with bacterial pneumonia, non-tuberculous mycobacterial (NTM) lung disease, or parasitic infections diagnosed during the same study period.

The study protocol was approved by the Ethics Committee of the Public Health Clinical Medical Center of Chengdu (Ethics Approval Number: YJ-K2024-75-01). The study was conducted in accordance with the Declaration of Helsinki.

Inclusion criteria were: (1) aged <18 years; (2) diagnosed with ATB or non-TB diseases.

Exclusion criteria included: (1) Human Immunodeficiency Virus (HIV) infection or Acquired Immune Deficiency Syndrome (AIDS); (2) non-ATB patients with a history of anti-TB therapy completion (≥6 months before enrollment) who had no current TB-related symptoms, negative etiological evidence within 3 months before enrollment, and stable imaging findings confirmed by at least 6 months of follow-up; (3) history of steroid treatment before hospitalization; (4) concurrent disease that could affect study results (e.g., cancer, autoimmune diseases); (5) incomplete clinical or laboratory data; (6) readmission to hospital (only patients with first hospitalization for diagnosis were included).

### 2.2 Data collection

Demographic data (age, sex, ethnicity, rural/urban area), clinical information (MTB exposure history, BCG vaccination status, TST results), and laboratory data were extracted from electronic medical records. Laboratory data were collected from the first test after hospital admission, including absolute counts of neutrophils, lymphocytes, platelets, CD4^+^ T cells, CD8^+^ T cells, and albumin levels. All laboratory tests were performed in the central laboratory of the Public Health Clinical Medical Center of Chengdu. Lymphocyte, neutrophil, and platelet counts were detected using a fully automated hematology analyzer (XN-9000, Sysmex, Japan); reference ranges for children/adolescents: lymphocytes 1.10–3.20 × 10^9^/L, neutrophils 1.80–6.30 × 10^9^/L, platelets 100–300 × 10^9^/L. (2) Albumin levels were measured via bromocresol green method on an automatic biochemical analyzer (Cobas 8000, Roche, Switzerland); reference range for children/adolescents: 35–55 g/L. (3) CD4+/CD8+ T cell counts were determined by flow cytometry (Navios, Beckman Coulter, USA) with monoclonal antibody panels; reference ranges for children/adolescents: CD4+ T cells 414–1,123 cells/uL, CD8+ T cells 238–874 cells/uL. The methods of calculating the LAP, logLAP, NLR, NAR, PLR, SII and PNI are as follows (8, 23, 24):


$$\mathrm{LAP}=\text{Lymphocyte}\;(\times 10^{9}/L)\times \text{Albumin}\;(g/L)$$

$$\text{logLAP}=\log_{e}\;(\text{Lymphocyte}\;(\times 10^{9}/L)\times \text{Albumin}\;(g/L))$$

$$\mathrm{NLR}=\text{Neutrocyte}\;(\times 10^{9}/L)÷\text{Lymphocyte}\;(\times 10^{9}/L)$$

$$\mathrm{NAR}=\text{Neutrocyte}\;(\times 10^{9}/L)÷\text{Albumin}\;(g/L)$$

$$\mathrm{PLR}=\text{Platelet}\;(\times 10^{9}/L)÷\text{Lymphocyte}\;(\times 10^{9}/L)$$

$$\begin{array}{l} \mathrm{SII}=\text{Neutrocyte}\;(\times 10^{9}/L)\times \text{Platelet}\;(\times 10^{9}/L)\\ ÷\text{Lymphocyte}\;(\times 10^{9}/L) \end{array}$$

$$\mathrm{PNI}=\text{Albumin}\;(g/L)+5\times \text{Lymphocyte}\;(\times 10^{9}/L)$$

To control for information bias in this retrospective design: (1) All data were extracted from electronic medical records using the FREE Electronic Data Capture System (version 2.3.1; Beijing FreeClinical Medical Technology Co., Ltd., Beijing, China), with double data entry and cross-validation performed by two independent researchers. (2) All blood samples were collected within 24 h of patient admission. (3) For key variables with missing data, we predefined handling strategies based on clinical reasoning. For BCG vaccination status, the National Immunization Program (NIP) of China mandates BCG vaccination for newborns, with a nationwide policy requiring mandatory administration within 24 h of birth. This has resulted in a high primary BCG vaccination coverage rate (over 98%) among the pediatric population (25). Therefore, patients with unknown vaccination status were included in the vaccinated group. For TST results, missing results were defaulted to negative. To assess the robustness of our findings, we performed a sensitivity analysis by excluding participants with missing TST results and re-evaluated the association between logLAP and ATB. (4) To minimize selection bias, we implemented strict inclusion and exclusion criteria and consecutively enrolled all eligible patients during the study period without arbitrary selective screening.

### 2.3 Statistical analyses

Continuous data were first tested for normality. Those not conforming to a normal distribution were expressed as median (interquartile range, IQR; Q1, Q3) and compared using the rank-sum test, while normally distributed continuous data were presented as mean ± standard deviation (SD) and analyzed using the independent samples t-test. Categorical data were described as frequencies and percentages (%) and compared using the chi-square test. For group comparisons, categorical variables were analyzed by the *χ^2^* test or Fisher’s exact test. Continuous variables with a normal distribution were analyzed by one-way ANOVA, while non-normally distributed data were compared using the Kruskal–Wallis H test.

The Receiver Operating Characteristic (ROC) curve and the area under the curve (AUC) were generated to compare the discriminatory ability of LAP with that of other biomarkers (NLR, NAR, PLR, PNI, SII) in predicting the risk of ATB infection. Breakpoint analysis was performed to assess the association between logLAP and ATB, with the lowest tertile (Q1) of logLAP as the reference group. Three models were established: Model 1 (crude model), Model 2 (adjusted for age, sex, ethnicity, and rural/urban area), and Model 3 (further adjusted for BCG vaccination, TST results, history of MTB exposure, CD4^+^ T cells, and CD8^+^ T cells). Breakpoint analysis was performed using the two-piece linear regression model with a data-driven approach, where the optimal breakpoint was identified by minimizing the residual sum of squares (RSS) of the regression model. Restricted cubic spline (RCS) analyses, with knots placed at the 5th, 35th, 65th, and 95th percentiles, were conducted to explore potential non-linear relationships after adjusting for covariates (age, sex, ethnicity, rural/urban area, BCG vaccination, TST results, history of MTB exposure, CD4^+^ T cells, and CD8^+^ T cells). Stratified analyses were conducted to assess the potential interactions of age, sex, ethnicity, rural/urban area, BCG vaccination, TST results, history of MTB exposure, CD4^+^ T cells, and CD8^+^ T cells on the association between logLAP and ATB infection.

Given that this study employed a retrospective design based on existing clinical data, no prospective sample size estimation was performed during the study design phase. The number of outcome events (ATB cases) observed in this study was 904, and the final multivariable logistic regression model included a total of nine covariates, yielding an events per variable (EPV) value of approximately 100.4, which exceeds the generally accepted minimum criterion (EPV ≥ 10) established in methodological studies. This high EPV value adequately ensures the stability of effect estimates, reduces the risk of overfitting, and guarantees sufficient detection power to identify clinically meaningful associations (26, 27). No post-hoc power analysis was conducted in this study, as it is mathematically redundant with the *p*-value and may lead to misleading interpretations (28). We have reported ORs with 95% CIs and verified the adequacy of the sample size through the sufficient EPV.

Statistical analyses were performed using R software^1^ (The R Foundation) and Free Statistics software version 2.3.1. *p* value < 0.05 was considered statistically significant.

## 3 Results

### 3.1 Study participants

**Figure 1:**
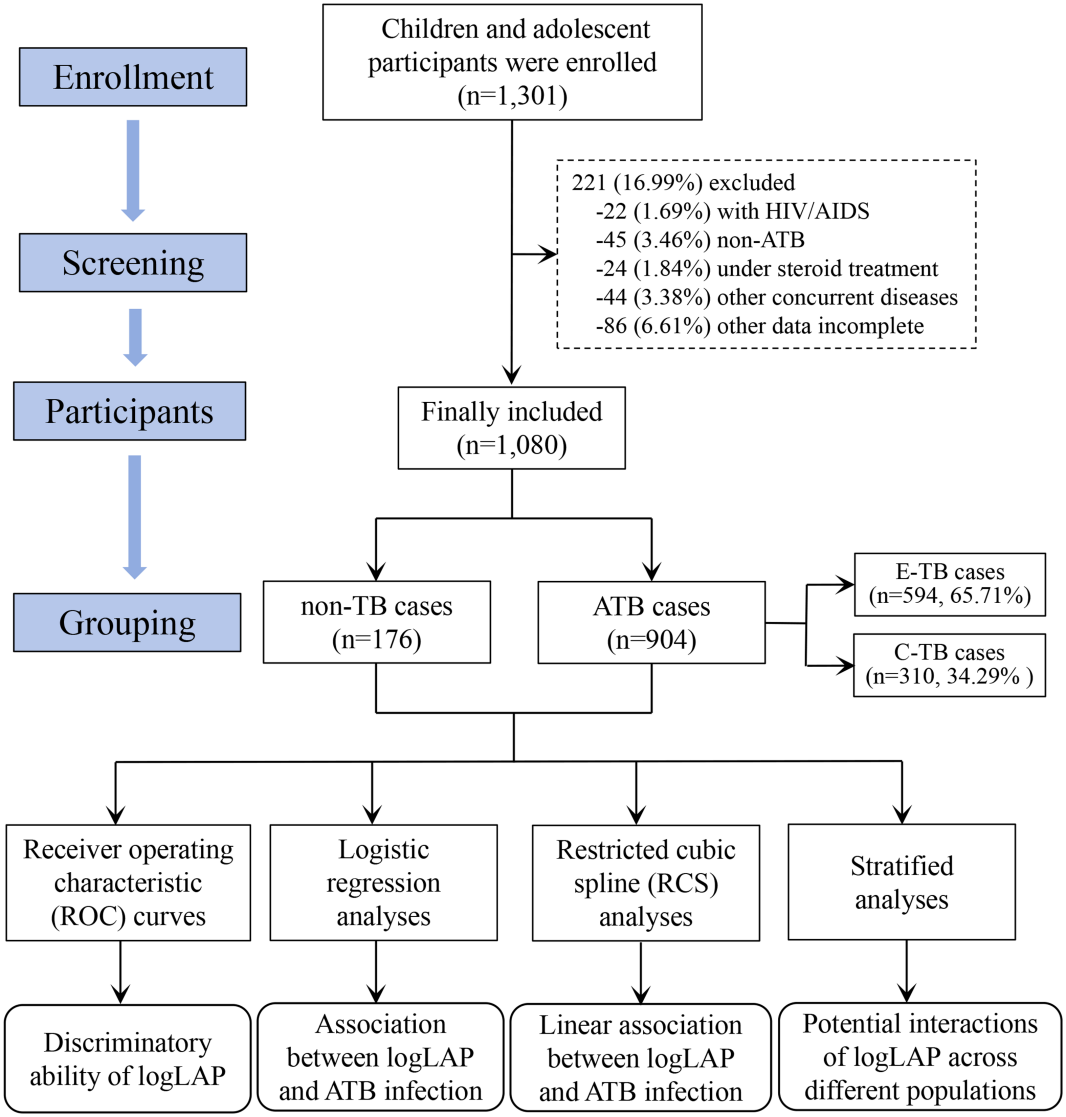
Flowchart of participants in this study. TB, tuberculosis; HIV, Human Immunodeficiency Virus; AIDS, Acquired Immune Deficiency Syndrome; ATB, active tuberculosis; E-TB, etiologically confirmed TB; C-TB, clinically diagnosed TB.

A total of 1,301 patients suspected of having TB were screened between April 2022 and December 2024. Of these, 221 were excluded based on the following criteria: concurrent HIV/AIDS (*n* = 22), non-ATB (*n* = 45; defined as a history of anti-TB therapy with no current symptoms, negative etiological evidence, and stable imaging findings), under steroid treatment (*n* = 24), other concurrent diseases (*n* = 44), and incomplete clinical data (*n* = 86). Consequently, a final cohort of 1,080 patients was enrolled for analysis, which included 904 patients ATB and 176 patients with non-TB conditions. The ATB group further was stratified into E-TB group (*n* = 594) and C-TB group (*n* = 310). The patient selection process was summarized in Figure 1.

### 3.2 Baseline characteristics

Table 1 presented the demographics and clinical characteristics of patients with ATB and non-TB controls. Significant differences were observed between the two groups in terms of age (*p* < 0.001), rural–urban residence (*p* = 0.006), ethnicity (*p* = 0.017), history of MTB exposure (*p* = 0.012), BCG vaccination status (*p* < 0.001), and TST results (*p* < 0.001). Compared with the non-TB group, the ATB group had a higher proportion of individuals aged 15–17 years (51.00% vs. 25.57%), those residing in rural areas (71.13% vs. 60.80%), non-Han ethnicity (74.12% vs. 65.34%), a history of MTB exposure (36.62% vs. 26.70%), no BCG vaccination (34.40% vs. 21.60%), and a positive TST result (60.84% vs. 21.02%).

**Table 1:** Demographics and clinical characteristics of participants.

Variables	Total (*n* = 1,080)	Non-TB (*n* = 176)	ATB (*n* = 904)	*p* value
Age, [years], *n* (%)				<0.001
0–7	115 (10.65)	34 (19.32)	81 (8.96)	
8–14	459 (42.50)	97 (55.11)	362 (40.04)	
15–17	506 (46.85)	45 (25.57)	461 (51.00)	
Sex, *n* (%)				0.328
Male	565 (52.31)	98 (55.68)	467 (51.66)	
Female	515 (47.69)	78 (44.32)	437 (48.34)	
Rural/urban, *n* (%)				0.006
Rural	750 (69.44)	107 (60.80)	643 (71.13)	
Urban	330 (30.56)	69 (39.20)	261 (28.87)	
Ethnicity, *n* (%)				0.017
Han	295 (27.31)	61 (34.66)	234 (25.88)	
Other	785 (72.69)	115 (65.34)	670 (74.12)	
Exposure status, *n* (%)				0.012
N	702 (65.00)	129 (73.30)	573 (63.38)	
Y	378 (35.00)	47 (26.70)	331 (36.62)	
BCG^a^, *n* (%)				<0.001
−	349 (32.31)	38 (21.60)	311 (34.40)	
+	731 (67.69)	138 (78.40)	593 (65.60)	
TST^b^, *n* (%)				<0.001
−	493 (45.65)	139 (78.98)	354 (39.16)	
+	587 (54.35)	37 (21.02)	550 (60.84)	

TB, tuberculosis; ATB, active tuberculosis; BCG, Bacillus Calmette-Guérin; TST, tuberculin skin test.

^a^Since newborns in China are required to receive BCG vaccination, patients with unknown vaccination status are included in the group with vaccination history (95 missing cases of BCG results, accounting for 8.80% of the total sample).

^b^Missing results are defaulted to negative (141 missing cases of TST results, accounting for 13.06% of the total sample). Considering the potential bias introduced by defaulting missing TST results to negative, we performed a sensitivity analysis after excluding the 141 participants with missing TST data. The baseline characteristics of the remaining 939 participants (Supplementary Table S1) were similar to those of the full cohort.

Significant differences were observed in laboratory data between the two groups (Table 2). The ATB group exhibited notably lower CD4^+^ T-cell counts (21.13% vs. 6.25%) and CD8^+^ T-cell counts (17.92% vs. 5.11%) compared to the non-TB group. Furthermore, several inflammatory and metabolic markers differed significantly, including the LAP (67.36 ± 41.63 vs. 88.04 ± 36.31, *p* < 0.001), NLR (2.60 vs. 2.03, *p* < 0.001), PLR (201.14 vs. 151.71, *p* < 0.001), SII (797.28 vs. 602.58, *p* < 0.001), and logLAP (4.07 ± 0.55 vs. 4.39 ± 0.47, *p* < 0.001). In contrast, NAR (*p* = 0.333) and PNI (*p* = 0.051) showed no statistically significant differences between the groups.

**Table 2:** Laboratory characteristics of participants.

Variables	Total (*n* = 1,080)	Non-TB (*n* = 176)	ATB (*n* = 904)	*p* value
CD4, cells/L, *n* (%)				<0.001
	699.63 ± 339.27	838.13 ± 331.63	672.66 ± 334.30	
≤414	202 (18.70)	11 (6.25)	191 (21.13)	
>414	878 (81.30)	165 (93.75)	713 (78.87)	
CD8, cells/L, *n* (%)				<0.001
	490.25 ± 272.41	643.20 ± 283.87	460.47 ± 260.02	
≤238	171 (15.83)	9 (5.11)	162 (17.92)	
>238	909 (84.17)	167 (94.89)	742 (82.08)	
LAP, Mean ± SD	70.73 ± 41.51	88.04 ± 36.31	67.36 ± 41.63	<0.001
NLR, Median (IQR)	2.51 (1.72, 3.94)	2.03 (1.24, 2.90)	2.60 (1.80, 4.03)	<0.001
NAR, Median (IQR)	0.09 (0.07, 0.13)	0.09 (0.06, 0.13)	0.10 (0.07, 0.13)	0.333
PLR, Median (IQR)	192.00 (138.02, 281.74)	151.71 (121.37, 212.07)	201.14 (144.08, 305.42)	<0.001
SII, Median (IQR)	751.13 (464.32, 1,334.41)	602.58 (378.49, 948.69)	797.28 (477.67, 1,395.28)	<0.001
PNI, Mean ± SD	41.39 ± 6.18	42.22 ± 6.33	41.22 ± 6.14	0.051
logLAP, Mean ± SD	4.12 ± 0.55	4.39 ± 0.47	4.07 ± 0.55	<0.001

TB, tuberculosis; ATB, active tuberculosis; LAP, lymphocyte-albumin product; NLR, Neutrophil-Lymphocyte ratio; NAR, Neutrophil-Albumin ratio; PLR, Platelet-Lymphocyte ratio; SII, Systemic immune-Inflammation index; PNI, Prognostic-Nutritional index; logLAP, logarithmic lymphocyte-albumin product.

### 3.3 Discriminatory ability of biomarkers

**Figure 2:**
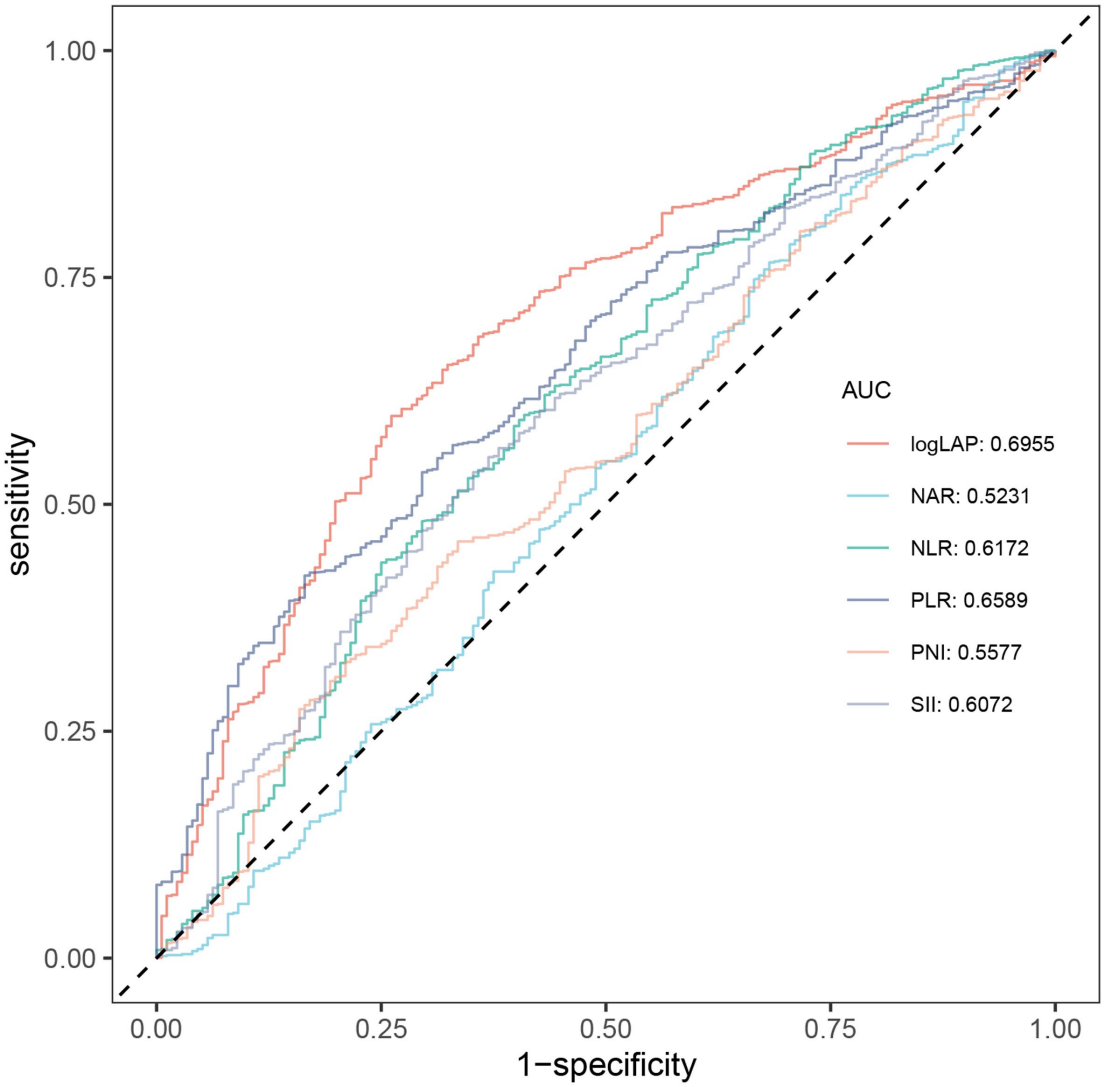
ROC curve analysis of biomarkers between ATB and non-TB. ROC, receiver operating characteristic; ATB, active tuberculosis; TB, tuberculosis; AUC, area under the curve; LAP, lymphocyte-albumin product; NAR, neutrophil-albumin ratio; NLR, neutrophil-lymphocyte ratio; PLR, platelet-lymphocyte ratio; PNI, prognostic-nutritional index; SII, systemic immune-inflammation index.

The area under the curve (AUC) was calculated to compare the discriminatory ability of LAP with that of other biomarkers (NLR, NAR, PLR, PNI, SII) in predicting the risk of ATB infection. Figure 2 presented ROC curves assessing the predictive performance of LAP and other biomarkers for ATB infection. LAP achieved the highest AUC (0.6955), followed by PLR (AUC = 0.6589), NLR (AUC = 0.6172), SII (AUC = 0.6072), PNI (AUC = 0.5577), and NAR (AUC = 0.5231). Table 3 further provided the sensitivity, specificity, Positive Predictive Value (PPV), Negative Predictive Value (NPV), accuracy, and Youden index of biomarkers between ATB and non-TB. The optimal clinical threshold for logLAP was determined using the Youden index, which maximizes the sum of sensitivity and specificity. At a logLAP cut-off of 4.23, the Youden index was 0.34, with a sensitivity of 60% and specificity of 74%.

**Table 3:** Discriminatory abilities of biomarkers between ATB and non-TB.

Variables	AUC (95% CI)	Cut-off values	Specificity	Sensitivity	Accuracy	PPV	NPV	Youden index
logLAP	0.6955 (0.6539–0.7370)	4.23	0.74	0.60	0.62	0.92	0.26	0.34
NAR	0.5231 (0.4733–0.5728)	0.07	0.32	0.77	0.69	0.85	0.21	0.08
NLR	0.6172 (0.5698–0.6646)	2.27	0.60	0.60	0.59	0.88	0.22	0.19
PLR	0.6589 (0.6181–0.6998)	225.12	0.84	0.42	0.49	0.93	0.22	0.26
PNI	0.5577 (0.5117–0.6037)	40.81	0.66	0.46	0.49	0.88	0.19	0.12
SII	0.6072 (0.5618–0.6525)	746.74	0.65	0.54	0.55	0.89	0.21	0.18

TB, tuberculosis; ATB, active tuberculosis; AUC, area under the curve; CI: confidence interval; LAP, lymphocyte-albumin product; NLR, Neutrophil-Lymphocyte ratio; NAR, Neutrophil-Albumin ratio; PLR, Platelet-Lymphocyte ratio; PNI, Prognostic-Nutritional index; SII, Systemic immune-Inflammation index; PPV: Positive Predictive Value; NPV: Negative Predictive Value.

### 3.4 Association between logLAP and ATB infection

To examine the association between logLAP and the risk of ATB infection, logLAP was further categorized into tertiles, and three multivariate models were constructed (see Table 4). In the unadjusted model (Model 1), OR was 0.28 (95% CI: 0.20–0.41, *p* < 0.001). After adjusting for age, sex, ethnicity, and rural/urban area in Model 2, compared with the first tertile Q1, the second tertile Q2 (OR = 0.60, 95% CI: 0.36–1.00, *p* = 0.048) and the third tertile Q3 (OR = 0.28, 95% CI: 0.17–0.45, *p* < 0.001) of logLAP showed a significant inverse association with ATB infection risk.

**Table 4:** Factors influencing the detection results of participants.

Variables	Model 1		Model 2		Model 3	
	OR (95%CI)	*p* value	OR (95%CI)	*p* value	OR (95%CI)	*p* value
logLAP	0.28 (0.20–0.41)	<0.001	0.39 (0.26–0.58)	<0.001		0.001
logLAP groups						
Q1 (*n* = 360)	1 (Ref)		1 (Ref)		1 (Ref)	
Q2 (*n* = 360)	0.52 (0.31–0.86)	0.011	0.60 (0.36–1.00)	0.048	0.76 (0.43–1.37)	0.361
Q3 (*n* = 360)	0.19 (0.12–0.31)	<0.001	0.28 (0.17–0.45)	<0.001	0.33 (0.18–0.59)	<0.001

Model 1: Crude.

Model 2: Adjust: Age, Sex, Ethnicity, Rural/Urban.

Model3: Adjust: Age, Sex, Ethnicity, Rural/Urban, BCG^a^, TST^b^, Exposure, CD4, CD8.

OR, odds ratio; CI, confidence interval; logLAP, logarithmic lymphocyte-albumin product; BCG, Bacillus Calmette-Guérin; TST, tuberculin skin test.

^a^Since newborns in China are required to receive BCG vaccination, patients with unknown vaccination status are included in the group with vaccination history (95 missing cases of BCG results, accounting for 8.80% of the total sample).

^b^Missing results are defaulted to negative (141 missing cases of TST results, accounting for 13.06% of the total sample). Considering the potential bias introduced by defaulting missing TST results to negative, we performed a sensitivity analysis after excluding the 141 participants with missing TST data. The association between logLAP and ATB remained consistent (Supplementary Table S2). In the fully adjusted model (Model 3), the third tertile of logLAP continued to show a significant inverse association with ATB (OR = 0.34, 95% CI: 0.17–0.67, *p* = 0.002), while the second tertile was not significant (OR = 0.55, 95% CI: 0.30–1.03, *p* = 0.062). These findings indicate that our primary results were not substantially affected by the handling of missing TST data.

Further adjustment for BCG vaccination status, TST results, history of MTB exposure, CD4^+^ T-cell count, and CD8^+^ T-cell count in Model 3 revealed that the association for the second tertile Q2 was no longer significant (OR = 0.76, 95% CI: 0.43–1.37, *p* = 0.361), while the inverse association remained significant for the third tertile Q3 (OR = 0.33, 95% CI: 0.18–0.59, *p* < 0.001).

Table 5 presented the data-driven post-hoc breakpoint analysis of the association between logLAP and ATB infection. The estimated breakpoint from this analysis was 3.48. When logLAP was ≥3.48, there was a significant inverse association with ATB infection (OR = 0.19, 95% CI: 0.12–0.30, *p* < 0.001), indicating that for each 1-unit decrease in logLAP, the risk of ATB infection increases by 81%. In contrast, when logLAP was <3.48, the association was substantially attenuated and no significant (OR = 1.01, 95% CI: 0.13–7.66, *p* = 0.991).

**Table 5:** Breakpoint analyses of the logLAP and ATB infection relationship.

Item	*n*	Breakpoint/OR (95%CI)	*p* value
logLAP < 3.48	126	1.01 (0.13–7.66)	0.991
logLAP ≥ 3.48	948	0.19 (0.12–0.30)	<0.001
Likelihood Ratio Test	–	–	0.158
Non-linear Test*1	–	–	0.190
Non-linear Test*2	–	–	0.004

OR, odds ratio; CI, confidence interval; logLAP, logarithmic lymphocyte-albumin product.

The breakpoint of logLAP = 3.48 was identified via a post-hoc data-driven approach (minimizing the residual sum of squares of the two-piece linear regression model), and the potential risk of overfitting was associated with this post-hoc threshold derivation.

**Figure 3:**
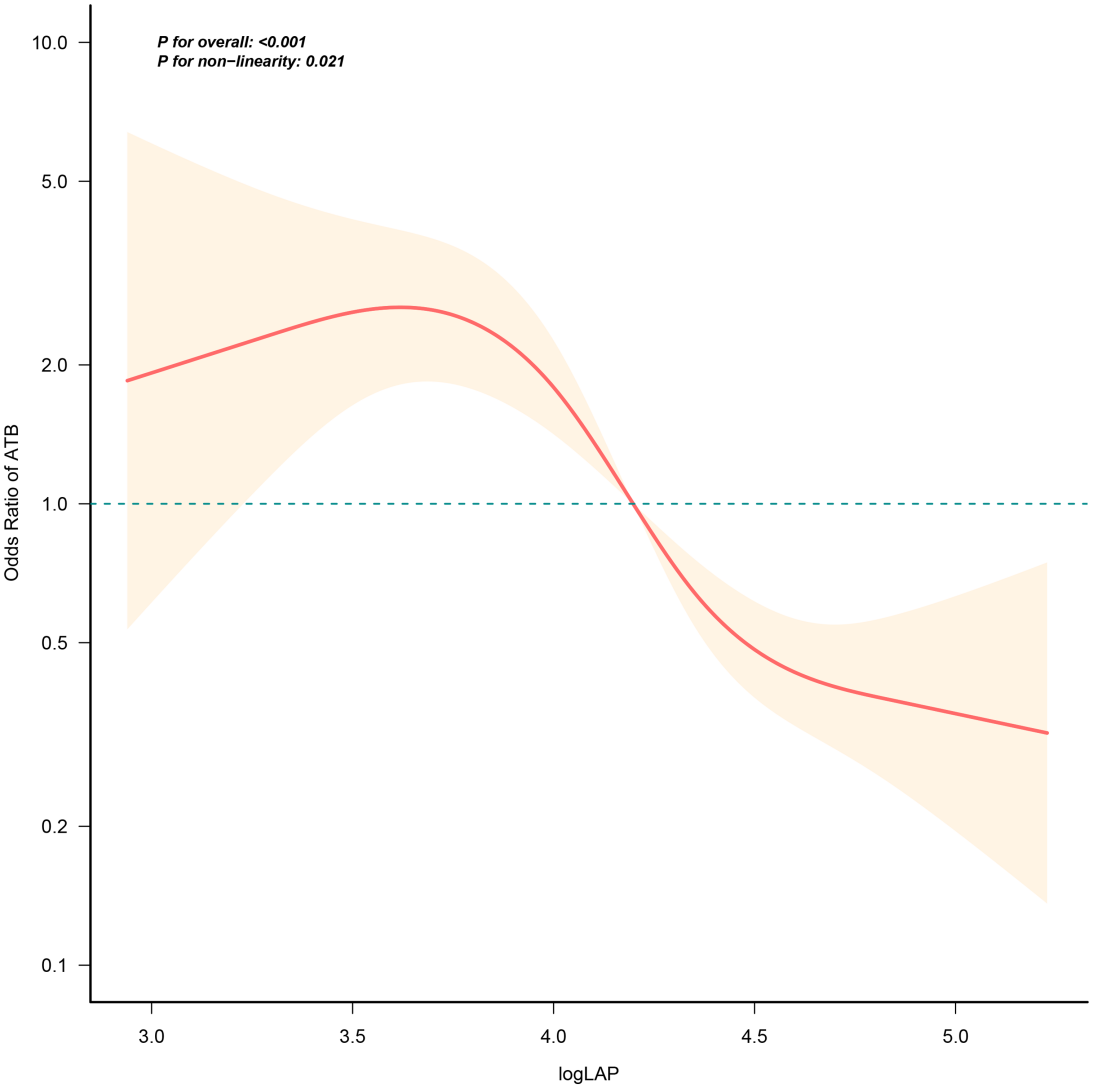
RCS curve of the relationship between logLAP and ATB infection. This RCS model was constructed with 4 knots (5th, 35th, 65th, 95th percentiles of logLAP distribution). The OR is represented by the red line, and the shaded part represents the 95% CI. RCS, restricted cubic spline; CI, confidence interval; logLAP, logarithmically transformed lymphocyte-albumin product; ATB, active tuberculosis.

In RCS model (knots placed at the 5th, 35th, 65th, and 95th percentiles), a significant non-linear relationship (*p* for non-linearity = 0.021) was observed (Figure 3). The RCS curve demonstrated an inflection point at approximately logLAP = 3.48, below which the risk of ATB increased sharply, and above which the risk declined steadily. This finding aligns with the breakpoint analysis, which identified a threshold at 3.48 (Table 5).

### 3.5 Potential interactions of logLAP across different populations

**Figure 4:**
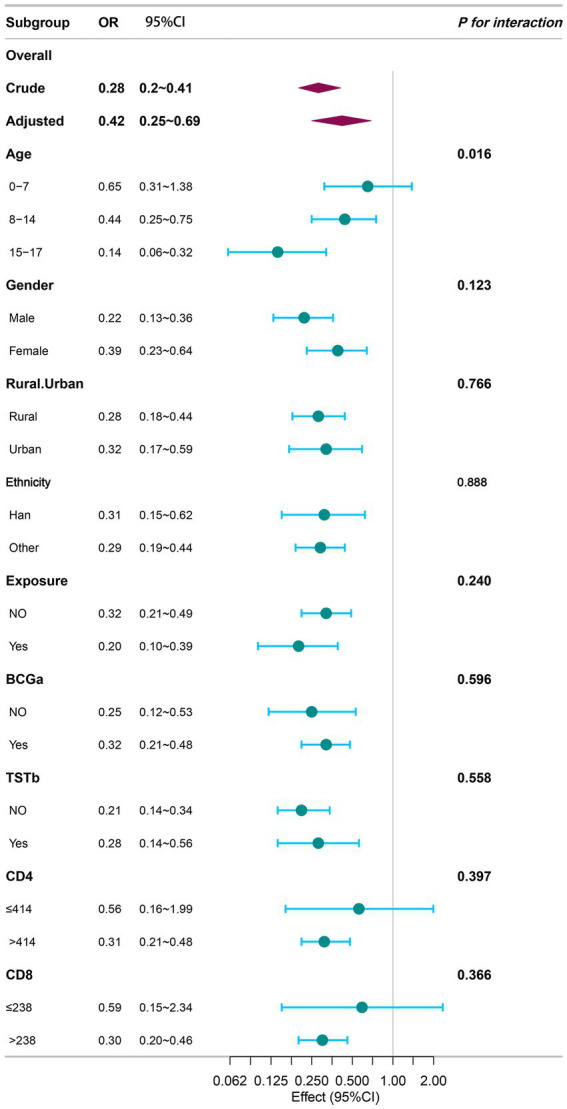
Stratified analyses for the association between logLAP and ATB infection. *p* for interaction indicated adjusting for age, sex, ethnicity, rural/urban, BCG^a^, TST^b^, exposure, CD4, CD8. OR: Odds ratio; CI, confidence interval; logLAP, logarithmically transformed lymphocyte-albumin product; ATB, active tuberculosis; BCG, Bacillus Calmette-Guérin; TST, tuberculin skin test. ^a^Since newborns in China are required to receive BCG vaccination, patients with unknown vaccination status are included in the group with vaccination history. ^b^Missing results are defaulted to negative.

Stratified analyses were conducted across 19 subgroups based on age, sex, rural/urban area, ethnicity, history of MTB exposure, BCG vaccination status, TST results, CD4^+^ T-cell count, and CD8^+^ T-cell count (Figure 4). The inverse association between logLAP and the risk of ATB infection remained consistent across all subgroups, except in the following: children aged 0–7 years, individuals with CD4^+^ T-cell counts ≤414 cells/μL, and those with CD8^+^ T-cell counts ≤238 cells/μL.

The association between logLAP and ATB varied significantly by age group (*p* for interaction = 0.016). The strongest inverse association was observed in the 15–17 years age group (OR = 0.14, 95% CI: 0.06–0.32), followed by the 8–14 years age group (OR = 0.44, 95% CI: 0.25–0.75). In contrast, the association was not statistically significant in children aged 0–7 years (OR = 0.65, 95% CI: 0.31–1.38). No significant interactions were found for sex, rural/urban area, ethnicity, history of MTB exposure, BCG vaccination status, TST results, CD4^+^ T-cell count, or CD8^+^ T-cell count (all *p* for interaction > 0.05).

## 4 Discussion

This retrospective study is the first to propose and validate the association between the immune-nutritional biomarker logLAP and ATB in children and adolescents, comparing its associative performance with existing inflammatory, immune, and nutritional biomarkers, and evaluating its discriminatory ability. The results demonstrated that logLAP levels were significantly lower in ATB patients compared to the non-TB control group. After adjusting for confounding factors (including demographic characteristics, BCG vaccination status, history of MTB exposure, and T-cell subsets), logLAP remained significantly negatively associated with ATB infection risk. In terms of discriminatory ability, LAP achieved AUC (0.6955) superior to traditional inflammatory ratios (NLR, PLR, SII), the immune-nutritional index (PNI), and the inflammatory-nutritional index (NAR). Breakpoint analysis identified a logLAP threshold of 3.48, below which the risk of ATB infection increased significantly. Stratified analyses further confirmed the consistency of the association of logLAP and ATB infection in most subgroups.

In Kissling’s study, NLR achieved an AUC of 0.82 when differentiating pediatric TB from lower respiratory tract infections (9). However, in a multicenter study by Cursi et al., the AUC of NLR for distinguishing pediatric ATB from other conditions (including latent infection and other infections) was 0.72 (10). Although the AUC values of NLR vary across different study designs and control populations, in the specific cohort and control settings of our study (bacterial pneumonia, non-TB mycobacterial pulmonary disease, parasitic infections), LAP (AUC = 0.6955) outperformed NLR (AUC = 0.6172). In the study by Rees et al., PLR was evaluated for predicting latent TB infection in adolescents and demonstrated poor performance (AUC = 0.56) (11). In the study by Yu et al., SII was used to distinguish active pulmonary TB from non-TB lung diseases, achieving an AUC of 0.62 (29). To date, no studies have been identified that applied SII for diagnosing TB in children. As a composite indicator integrating nutrition and immunity, PNI has been widely applied in oncology and rheumatoid arthritis. In TB research, Tan et al. demonstrated that PNI was an independent predictor of prognosis in adult pulmonary TB patients, achieving an AUC of 0.862 for predicting unfavorable outcomes (13). Similarly, Lu et al. showed that PNI serves as an independent protective factor against disease severity in active pulmonary TB, and when combined with BMI, PLR, and traditional clinical indicators, it achieved an AUC of 0.701 for diagnosing severe TB (12). However, due to differences in immunological and physiological characteristics between children and adults, findings from adult studies cannot be directly extrapolated to pediatric populations (30). To date, no studies have specifically examined the association between PNI and ATB in children and adolescents. In our cohort, PNI demonstrated a diagnostic AUC of only 0.5577 for ATB in children and adolescents, indicating limited diagnostic value. This may be attributed to PNI’s additive calculation of albumin and lymphocytes, which assumes that nutritional and immune statuses contribute independently to TB risk, thereby failing to capture the interdependence between immune defense and albumin-related nutritional support. Notably, immuno-nutritional differences between children and adults may influence the performance of logLAP and other biomarkers. Children and adolescents have immature immune systems and distinct albumin metabolism compared to adults (30, 31). These age-related characteristics may alter the synergistic effect between lymphocyte counts and albumin levels, leading to variations in the association between logLAP and ATB across different age groups. Such developmental factors may affect the performance of composite biomarkers such as the PNI, which could explain its relatively limited diagnostic efficacy in our pediatric population, in contrast to its more pronounced effectiveness reported in adults. This aligns with our finding that logLAP showed no significant association with ATB in children aged 0–7 years. In contrast, LAP integrates lymphocyte count-a key cellular component of immunity, and albumin-a marker inversely associated with nutrition and inflammation, through direct multiplication. This multiplicative formulation captures the synergistic interplay between nutrition and immunity more comprehensively than PNI’s additive calculation, while being more straightforward to compute.

In the breakpoint analysis, a logLAP threshold of 3.48 was identified. However, as this value was derived from a data-driven post-hoc analysis, it may be subject to overfitting. To enhance clinical applicability, we determined the optimal diagnostic threshold using the Youden index, which yielded a logLAP cut-off of 4.23. This value corresponds to a sensitivity of 60% and specificity of 74%, indicating moderate diagnostic performance. While the breakpoint of 3.48 reflects a biological inflection point for immune-nutritional response mounted by the host against MTB infection, the Youden-derived threshold of 4.23 is more suitable for clinical decision-making, as it balances sensitivity and specificity in differentiating ATB from non-TB conditions. In the stratified analyses, the absence of a significant correlation between logLAP and ATB in children aged 0–7 years may be attributable to the immature immune system and distinct nutritional metabolic characteristics of young children, whose lymphocyte subsets and albumin synthesis remain in developmental stages (31). Consequently, the synergistic depletion of immune and nutritional status induced by MTB infection may not be effectively captured by the logLAP computational model in this population. In the immunocompromised subgroup, the lack of a significant association between logLAP and ATB stems from severe cellular immune deficiency. CD4+ T cells are central to the adaptive immune response against MTB, responsible for activating macrophages, promoting B cell antibody production, and regulating immune memory (32). CD8+ T cells directly kill MTB-infected cells and secrete antiviral/anti-inflammatory cytokines (33). When CD4+ or CD8+ T cell counts are substantially reduced, the cellular immune response against MTB becomes nearly paralyzed, and lymphocyte counts no longer reflect the host’s immune status against MTB. Therefore, compromised immune function affects the relationship between logLAP and ATB.

The inverse correlation between logLAP and the risk of ATB is supported by a plausible biological basis. MTB infection triggers a systemic inflammatory response, leading to lymphocytopenia due to lymphocyte migration to infection sites and increased apoptosis (16). Mechanistically, MTB promotes the apoptosis of CD4+ and CD8+ T lymphocytes—cells crucial for anti-tuberculosis immunity—through the Fas/FasL pathway and the release of interferon gamma (IFN-γ) (34, 35). Studies have shown that markers such as CD38, human leukocyte antigen-DR isotype (HLA-DR) on the surface of IFN-γ+ CD4+ T cells, and intracellular Ki-67 are closely correlated with MTB bacterial load and treatment response (36). Concurrently, serum albumin, a classic indicator of systemic nutritional status and inflammatory severity, is often reduced in ATB patients, as chronic inflammation impairs hepatic albumin synthesis, resulting in hypoalbuminemia (37). This hypoalbuminemia is not merely due to malnutrition but rather results from the suppression of albumin synthesis in the liver caused by chronic inflammation. Pro-inflammatory cytokines such as interleukin-6 (IL-6) and tumor necrosis factor-alpha (TNF-α) downregulate albumin gene expression at the transcriptional level, a mechanism well-established in chronic infections and inflammatory diseases (38, 39). The combined decline in lymphocyte count and albumin count in ATB patients contributes to a reduced LAP value. Therefore, the production of lymphocyte and albumin may amplify this synergistic effect. This is consistent with our finding that logLAP was significantly lower in the ATB group compared to the non-TB group. In stratified analyses, the association between logLAP and ATB was not significant in subgroups with low CD4^+^ and CD8^+^ T-cell counts. This finding indirectly underscores the link between logLAP and cellular immune function; specifically, when T-cell counts are reduced, lymphocytes contribute less to the logLAP value, which in turn impairs its ability to distinguish between cases and controls. Conversely, the association was stronger in older children and adolescents, which may be attributed to their more mature immune systems that respond to MTB infection in a manner more similar to adults (30).

The primary strength of this study is its innovative approach, as it is the first to propose and systematically evaluate the composite biomarker LAP/logLAP for its clinical associative performance and discriminatory ability in children and adolescents with ATB. A comprehensive evaluation was conducted through confounding factor adjustments, ROC comparisons, RCS analyses, and extensive stratified analyses, thoroughly assessing the association between logLAP and ATB, and subgroup stability of logLAP in relation to ATB. Moreover, this study included a large sample size of over 1,000 children and adolescents, which enhances the reliability of the findings. In terms of discriminatory ability, logLAP achieved AUC superior to traditional inflammatory ratios (NLR, PLR, SII), the immune-nutritional index (PNI), and the inflammatory nutritional index (NAR). Notably, an AUC of 0.6955 (logLAP specificity 0.74, sensitivity 0.60) indicates only moderate discriminatory ability. Therefore, logLAP should be positioned as an adjunctive indicator, providing supplementary reference for clinical assessment alongside other diagnostic methods. Nevertheless, this study has several limitations. First, the control group consisted of patients with other infectious diseases rather than healthy individuals or non-infectious controls, which may have independently affected inflammatory and immunological indicators, potentially influencing the diagnostic performance of logLAP. Second, as a retrospective single-center study conducted in a specialized infectious disease hospital, selection and information bias were unavoidable; the study cohort was biased toward severe or complex cases, which may have amplified group differences and overestimated the discriminative value of logLAP in mild or early-stage disease; internal validation methods were not performed, which may limit the direct extrapolation of the study results for immediate clinical application. Moreover, residual confounding cannot be excluded, as some nutritional and socioeconomic factors were not fully adjusted for, and age heterogeneity was observed, with no significant association detected in younger children. Third, the retrospective design precluded causal inference, and the lack of external validation from multicenter or geographically diverse cohorts limits the generalizability of the findings. Fourth, missing data on BCG vaccination and TST results were handled using simplified assumptions, which may have introduced misclassification bias. Fifth, logLAP showed only moderate diagnostic accuracy (AUC = 0.6955), indicating it cannot serve as a standalone test and requires combination with other clinical or laboratory markers. Finally, no prospective validation was performed to evaluate its predictive value for incident TB in at-risk populations, restricting its application in clinical screening. Future large-sample, age-stratified, multicenter studies with well-defined healthy or non-infectious controls are warranted. Prospective designs and external validation are needed to confirm the clinical utility of logLAP for pediatric TB screening and early diagnosis, while standardized data collection can help minimize bias related to missing information.

## 5 Conclusion

In summary, this study is the first to propose and demonstrate that the logLAP, a biomarker derived from routine blood test parameters, is significantly and inversely associated with the risk of ATB infection in children and adolescents aged ≥8 years with normal or moderately impaired immune function. Compared with traditional inflammatory ratios (NLR, PLR, SII), the immune-nutritional index (PNI), and the inflammatory-nutritional index (NAR), logLAP exhibits moderate discriminatory ability in differentiating ATB from non-TB diseases, and its non-linear association with ATB infection with an inflection point at 3.48. Notably, the AUC value of logLAP may have been underestimated due to the heterogeneity of the non-TB control group in this study. As a composite indicator, logLAP comprehensively captures the concurrent immune depletion and nutritional imbalance in the host under MTB infection. Given its simple calculation and low cost, logLAP is suitable as an adjunctive indicator in for children and adolescents ATB resource-limited regions or for identifying high-risk individuals, thereby facilitating early diagnosis and intervention.

## Data Availability

The raw data supporting the conclusions of this article will be made available by the authors, without undue reservation.

## Footnotes

1
http://www.R-project.org

## Glossary

logLAP: Logarithmic Lymphocyte-Albumin Product
ATB: Active Tuberculosis
TB: Tuberculosis
LAP: Lymphocyte-Albumin Product
ROC: Receiver Operating Characteristic
NLR: Neutrophil-Lymphocyte Ratio
NAR: Neutrophil-Albumin Ratio
PLR: Platelet-Lymphocyte Ratio
SII: Systemic Immune-Inflammation Index
PNI: Prognostic-Nutritional Index
AUC: Area Under the Curve
OR: Odds Ratio
CI: Confidence Interval
RSS: Residual Sum of Squares
RCS: Restricted Cubic Spline
AFB: Acid-Fast Bacillus
MTB: *Mycobacterium Tuberculosis*
TST: Tuberculin Skin Test
IGRAs: Interferon-Gamma Release Assays
BCG: Bacillus Calmette–Guérin
CBC: Complete Blood Count
HIV: Human Immunodeficiency Virus
AIDS: Acquired Immune Deficiency Syndrome
E-TB: Etiologically Confirmed TB
C-TB: Clinically Diagnosed TB
NTM: Non-Tuberculous Mycobacterial
NIP: National Immunization Program
IQR: Interquartile Range
SD: Standard Deviation
Q1: First Tertile
Q2: Second Tertile
Q3: Third Tertile
PPV: Positive Predictive Value
NPV: Negative Predictive Value
IFN-γ: Interferon gamma
HLA-DR: Human Leukocyte Antigen – DR isotype
IL-6: Interleukin-6
TNF-α: Tumor Necrosis Factor-alpha
BMI: Body Mass Index
