# Supplementary material for: Association of logarithmic lymphocyte-albumin product with active tuberculosis in children and adolescents
Source: Front Nutr. 2026 Apr 14;13:1781667. doi: 10.3389/fnut.2026.1781667 (PMC13123255; doi:10.3389/fnut.2026.1781667)
Supplement: Supplementary file 1 [file Table_1.DOCX]

Table S1 Demographics and clinical characteristics of participants.

| **Variables** | **Total**  **(n = 1,080)** | **non-TB**  **(n = 176)** | **ATB**  **(n = 904)** | ***P* value** |
| --- | --- | --- | --- | --- |
| **Age, [years], n (%)** |  |  |  | < 0.001 |
| 0-7 | 115 (10.65) | 34 (19.32) | 81 (8.96) |  |
| 8-14 | 459 (42.50) | 97 (55.11) | 362 (40.04) |  |
| 15-17 | 506 (46.85) | 45 (25.57) | 461 (51.00) |  |
| **Sex, n (%)** |  |  |  | 0.328 |
| Male | 565 (52.31) | 98 (55.68) | 467 (51.66) |  |
| Female | 515 (47.69) | 78 (44.32) | 437 (48.34) |  |
| **Rural/ Urban, n (%)** | |  |  | 0.006 |
| Rural | 750 (69.44) | 107 (60.80) | 643 (71.13) |  |
| Urban | 330 (30.56) | 69 (39.20) | 261 (28.87) |  |
| **Ethnicity, n (%)** | |  |  | 0.017 |
| Han | 295 (27.31) | 61 (34.66) | 234 (25.88) |  |
| Other | 785 (72.69) | 115 (65.34) | 670 (74.12) |  |
| **Exposure status, n (%)** | |  |  | 0.012 |
| N | 702 (65.00) | 129 (73.30) | 573 (63.38) |  |
| Y | 378 (35.00) | 47 (26.70) | 331 (36.62) |  |
| **BCG^a^, n (%)** | |  |  | < 0.001 |
| - | 349 (32.31) | 38 (21.60) | 311 (34.40) |  |
| + | 731 (67.69) | 138 (78.40) | 593 (65.60) |  |
| **TST^b^, n (%)** | |  |  | < 0.001 |
| - | 352 (37.49) | 131 (77.98) | 221 (28.66) |  |
| + | 587 (62.51) | 37 (22.02) | 550 (71.33) |  |

TB, tuberculosis; ATB, active tuberculosis; BCG, Bacillus Calmette-Guérin; TST, tuberculin skin test.

^a^: Since newborns in China are required to receive BCG vaccination, patients with unknown vaccination status are included in the group with vaccination history (95 missing cases of BCG results, accounting for 8.80% of the total sample).

^b^: Excluding the 141 participants with missing TST data.

TABLE S2 Factors Influencing the Detection Results of participants.

| **Variables** | **Model 1** | | **Model 2** | | **Model 3** | |
| --- | --- | --- | --- | --- | --- | --- |
|  | **OR (95%CI)** | **P value** | **OR (95%CI)** | **P value** | **OR (95%CI)** | **P value** |
| logLAP | 0.28 (0.20-0.41) | <0.001 | 0.99 (0.99-1.00) | 0.003 | 0.38 (0.20-0.74) | 0.004 |
| logLAP groups |  |  |  |  |  |  |
| Q1 (n=313) | 1(Ref) |  | 1(Ref) |  | 1(Ref) |  |
| Q2 (n=313) | 0.52 (0.31-0.86) | 0.011 | 0.56 (0.33-0.93) | 0.026 | 0.55 (0.30-1.03) | 0.062 |
| Q3 (n=313) | 0.19 (0.12-0.31) | <0.001 | 0.26 (0.16-0.43) | <0.001 | 0.34 (0.17-0.67) | 0.002 |

Model 1: Crude.

Model 2: Adjust: Age, Sex, Ethnicity, Rural/ Urban.

Model3: Adjust: Age, Sex, Ethnicity, Rural/ Urban, BCG^a^, TST^b^, Exposure, CD4, CD8.

OR, odds ratio; CI, confidence interval; logLAP, logarithmic Lymphocyte-Albumin product; BCG, Bacillus Calmette-Guérin; TST, tuberculin skin test.

a: Since newborns in China are required to receive BCG vaccination, patients with unknown vaccination status are included in the group with vaccination history (95 missing cases of BCG results, accounting for 8.80% of the total sample).

b: Excluding the 141 participants with missing TST data.
